# The place of S-ketamine in fibromyalgia treatment (ESKEFIB): study protocol for a prospective, single-center, double-blind, randomized, parallel-group, dose-escalation controlled trial

**DOI:** 10.1186/s13063-021-05814-4

**Published:** 2021-11-27

**Authors:** Zuzana Javorcikova, Michel Dangoisse, Stéphane Nikis, Jean-Paul Lechat, Aline Gillain, Jean-François Fils, Philippe Van der Linden

**Affiliations:** 1 Grand Hôpital de Charleroi, Site Notre-Dame, Grand Rue 3, B-6000 Charleroi, Belgium; 2 Grand Hôpital de Charleroi, Site Sainte-Thérèse, Rue Trieu Kaisin 134, 6061 Charleroi, Belgium; 3 Ars Statistica, Boulevard des Archers 40 B-1400, Nivelles, Belgium; 4 Université Libre de Bruxelles, Brussels, Belgium

**Keywords:** S-ketamine, Fibromyalgia, Chronic pain, Treatment, N-Methyl-D-aspartate receptor

## Abstract

**Background:**

Fibromyalgia is a chronic multidimensional pain disease with no curative treatment currently available. Its management relies on a multimodal approach involving pharmacologic and non-pharmacologic elements. Because a suggested factor in its etiology is a central sensitization phenomenon involving the N-methyl-D-aspartate receptor (NMDAR), NMDAR antagonists have been proposed as a treatment target. Ketamine and its levogyre form, S-ketamine, have been used to treat chronic pain for many years without consensus about their therapeutic efficiency. We aim to assess the efficacy of S-ketamine as a co-treatment for fibromyalgia.

**Methods:**

This prospective, randomized, single-center, double-blind, parallel-group, dose-escalation trial will compare a co-treatment with S-ketamine (intervention) to a control treatment without S-ketamine (control). It will consist of two successive cohorts with 2:1 randomization ratio (S-ketamine at two different doses: control) with 105 participants in each cohort. The protocol follow-up time will be 12 weeks, including 3 visits for the treatment (week 0, week 2, and week 4) and 3 visits for follow-up (week 6, week 9, and week 12). Our primary outcome, pain relief and/or better patient function, will be assessed with the Brief Pain Inventory questionnaire. The statistical analysis will be performed on an intention-to-treat basis. If the primary outcome is reached at the end of follow-up in the first cohort with low-dose S-ketamine (0.2 mg/kg), the trial will end. If not, the trial will continue with the second cohort and high-dose S-ketamine (0.4 mg/kg).

**Discussion:**

The challenge of our trial is the inclusion of a large number of participants in comparison to other trials involving ketamine or S-ketamine infusions for chronic pain management. The originality of our protocol is to include functionality in addition to pain relief as a primary outcome because these two endpoints are not linked in a linear way. For some patients, functional status is more important than pain relief.

**Trial registration:**

EudraCT reference: 2020-000473-25, ClinicalTrials.gov: NCT04436250, first posted June 18, 2020; last updated July 21, 2020. Protocol version 2.2 issued on September 30, 2020, after a revision by the ethics committee. https://clinicaltrials.gov/ct2/show/NCT04436250

**Supplementary Information:**

The online version contains supplementary material available at 10.1186/s13063-021-05814-4.

## Background

### Rationale

Chronic pain is a multidimensional syndrome affecting 17–44% of the population of western countries [1, 2]. Fibromyalgia has been classified in the International Classification of Diseases as a primary chronic pain disease [3], with specific diagnostic criteria established by the American College of Rheumatology (ACR) [4]. Its prevalence in western countries varies from 0.7 to 3.3% [5, 6]. The condition leads to progressive deterioration in quality of life, as well as a reduction in relational and functional capacity, resulting in major personal and societal cost [4–6]. No curative treatment is currently available, and recent recommendations advise a multimodal approach combining pharmacologic and non-pharmacologic therapies with the objective of reducing pain and improving quality of life [7].

A central sensitization phenomenon has been proposed as one of the etiological mechanisms in fibromyalgia [8, 9]. This process has been defined by the International Association for the Study of Pain (IASP) as an “increased responsiveness of nociceptive neurons in the central nervous system to their normal or subthreshold afferent input” [10]. The N-methyl-D-aspartate receptor (NMDAR), located in the central nervous system, is a candidate factor in this process because its activation plays a major role in cognition, chronic pain, and neuroplasticity. Ketamine, a drug used for anesthesia since the 1970s, acts as a noncompetitive antagonist to NMDAR [11].

Ketamine has been hypothesized to provoke a “reset of the central nervous system,” reversing the deleterious effects of central sensitization. This drug thus has been proposed as a co-treatment for chronic pain involving central sensitization phenomena, such as fibromyalgia [11]. Ketamine exists as two stereoisomers: R-ketamine and S- ketamine. S-ketamine is about two to four times more potent than the racemic mixture, enabling lower dosage with the S- form for equianalgesic effects [12]. The advantage with the S-ketamine stereoisomer is in limiting psychotropic effects, fatigue, and temporary cognitive impairment compared with using the racemic mixture [13]. These characteristics are especially interesting for ambulatory administration.

Ketamine has been used in chronic pain treatment for as long as 20 years with various dosage and administration modalities, and consensus guidelines for its use have been established [11]. However, in a recent meta-analysis, Orhurhu et al. [14] observed that most studies assessing its efficacy are at high risk of bias, including small populations of patients. Many questions remain unanswered about the optimal dose of ketamine, frequency of administration, which diseases to target, and the real impact on patient functional status, in addition to pain relief. The authors of that meta-analysis call for new prospective randomized studies to address these questions [14], but to date, no recent large-scale study to our knowledge includes patients with fibromyalgia.

### Objectives

Our hypothesis is that S-ketamine, through its NMDAR antagonist properties, would improve the quality of life (pain reduction and/or functionality) of patients diagnosed with a fibromyalgia syndrome according to the ACR 2016 criteria and who exhibit the characteristics of a central sensitization phenomenon (Central Sensitisation Inventory (CSI) > 40) [15, 16]. If the results support our hypothesis, S-ketamine could have a role as a co-treatment for fibromyalgia in patients with central sensitization when recommended treatments remain unsatisfactory.

Our primary objective is to evaluate whether S-ketamine used as a co-treatment in chronic pain management will result in better pain control and/or in improvement of functionality in patients with fibromyalgia. We specifically target this double objective because pain relief and improved function are only partially linked: better pain relief does not always lead to improved functionality, and some patients prioritize a gain in function despite maintaining the same level of pain. In fact, studies have not been able to demonstrate a linear relationship between pain relief and gain of function [17, 18].

Our secondary objectives are to assess how S-ketamine used as a co-treatment affects quality of life, satisfaction, and emotional status, all of which are important in chronic pain syndromes.

## Methods/design

The trial respects the Guidelines ICH E6 for Good Clinical Practice (01/07/2002), the principles of the Helsinki declaration, the ICH E2A Guidelines (Clinical Safety Data Management: Definitions and Standards for Expedited Reporting), the European Union Clinical Trial Directive (2001/20/EC), and all Belgian legislation to maximize participant safety and protection.

Relative to the May 7, 2004, Belgian legislation concerning experimentation on human subjects, a no-fault insurance has been subscribed in the name of the Grand Hôpital de Charleroi, Belgium (GHdC).

### Trial design

**Fig. 1:**
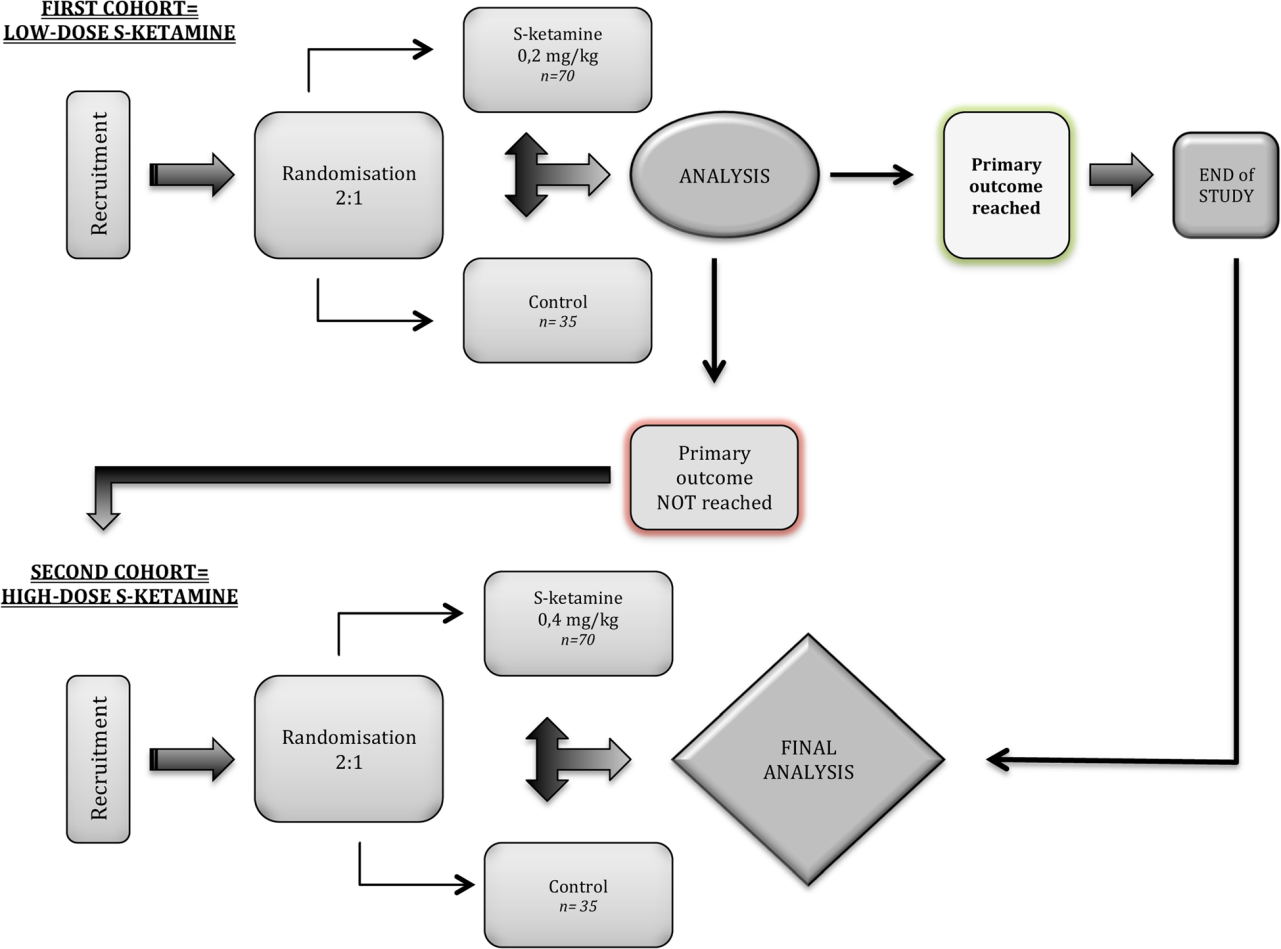
Trial design

This is a prospective, randomized, monocentric, double-blind, parallel-group, dose-escalation superiority study comparing an analgesic treatment including S-ketamine (intervention) to the same analgesic treatment without S-ketamine (control) (Fig. 1). The study design was inspired by Komen et al [19]. The dose-escalation design calls for a model with two successive cohorts, the first for comparing low-dose S-ketamine with the control treatment and the second cohort for comparing high-dose S-ketamine with the same control treatment.

This clinical trial is reported according to the Standard Protocol Items: Recommendations for Interventional Trials (SPIRIT) (see additional file 1).

#### Study setting

This study will be performed in the pain clinic of the GHdC, site Sainte-Thérèse, Charleroi, Belgium.

### Treatment

**Fig. 2:**
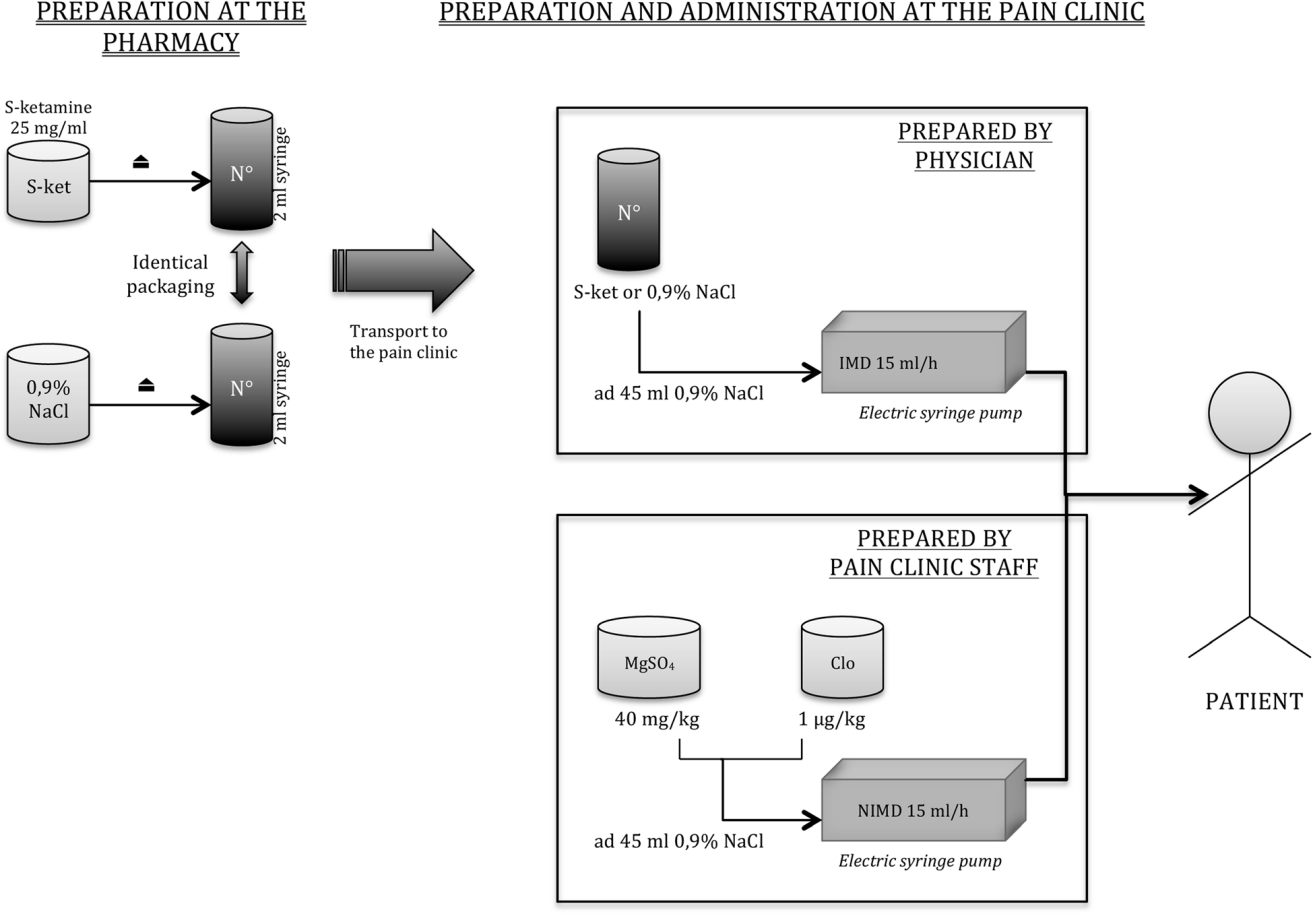
Treatment preparation steps. ⏏, laminar flow hood; S-ket, S-ketamine; N°, recognition number; MgSO_4_, magnesium sulfate; Clo, clonidine; IMD, Investigational Medication Drug; NIMD, non-investigational medication drug

The intravenous route has been chosen for the administration of all drugs and provides 100% bioavailability and better reproducibility [11]. The different preparation steps are summarized in Fig. 2.

#### S-ketamine: investigational medication drug

Because this study is a dose-escalation type of study, we have chosen two dose regimens: low-dose S-ketamine at 0.2 mg/kg and high-dose S-ketamine at 0.4 mg/kg, both in kilograms of corrected ideal body weight according to the Lorentz formula [20]. These doses are subanesthetic and suitable and secure for ambulatory administration [21, 22]. They are consistent with equianalgesic ketamine doses recommended for pain treatment, as well as our current clinical practice [11, 14, 23].

S-ketamine will be prepared by the GHdC pharmacy, which will condition ampoules of S-ketamine (25 mg/ml) under a laminar flow hood into syringes of 2 ml containing 25 mg/ml of S-ketamine. Each syringe will have identical packaging with a recognition number linked to the randomization. These syringes will be sent to the pain clinic, where the responsible physician will prepare the right dosage of S-ketamine according to the patient’s weight and dilute it in physiological saline (0.9% NaCl) to obtain a volume of 45 ml. The product will be administered intravenously over 3 h (15 ml/h) with an electronic syringe pump.

#### Placebo

The placebo, physiological saline (0.9% NaCl), also will be prepared by the GHdC pharmacy, conditioned in syringes of 2 ml with identical packaging to those containing S-ketamine. Each syringe will have a recognition number linked to the randomization. These syringes will be sent to the pain clinic, where the responsible physician will prepare the placebo as if it were S-ketamine, diluting it in physiological saline (0.9% NaCl) to obtain a volume of 45 ml. This solution will be administered intravenously over 3 hours (15 ml/h) with an electronic syringe pump.

#### Control treatment: non-investigational medication drugs

All participants will receive a control treatment of magnesium sulfate 40 mg/kg, for antinociceptive effects [24, 25], and clonidine 1 μg/kg, an alpha-2 agonist acknowledged for analgesic effects. These doses are suitable for ambulatory treatment [26, 27]. Both drugs are used to achieve analgesia, so that even patients in the control group will experience an analgesic effect. The use of clonidine also is expected to alleviate any possible psychomimetic influence of S-ketamine. In fact, clonidine is among the low-dose benzodiazepines and alpha-2 agonists that are recommended for preventing or aborting ketamine-induced hallucinations or dissociation [11].

Pain clinic staff will prepare the control treatment according to the usual procedure. Magnesium sulfate 40 mg/kg (from 3 g/10 ml ampoules) and clonidine 1 μg/kg (from 150 μg/ml ampoules) will be administered together, diluted in physiological saline (NaCl 0.9%) to obtain a volume of 45 ml. The product will be administered intravenously over 3 h (15 ml/h) with an electronic syringe pump.

#### Regimen

Each patient will simultaneously receive an infusion of the control treatment and an infusion of the test drug (S-ketamine) or placebo, given via two different electric syringe pumps over the 3-h time period. Each participant will receive the treatment three times at 2-week intervals, which is consistent with our current practice and findings in the literature [14].

The trial’s follow-up time is 12 weeks and involves three visits for the treatment (week 0, week 2, and week 4) and three visits for follow-up (week 6, week 9, and week 12) (Table 1).

**Table 1:** Summary of assessments, patient’s timeline

	Study period							
	Enrolment	Allocation	Post-allocation					Close-out
Timepoint	***T-2 weeks***	t-2 weeks ➔ t0	***T***_***0***_	***T+ 2 weeks***	***T + 4 weeks***	***T***_***+***_ ***6 weeks***	***T + 9 weeks***	***T + 12 weeks***
**Enrolment:**								
**Eligibility screen**	X							
**Informed consent**	X							
***Pregnancy test or electrocardiogram if needed***	X							
**Allocation**		X						
**Interventions:**								
***Treatment administration***			X	X	X			
***Follow-up consultation***						X	X	X
**Assessments:**								
***Central Sensitization Inventory (CSI)***			X					X
***EQ5D-5 L***			X					X
***Hospital Anxiety and Depression Scale (HADS)***			X					X
***Brief Pain Inventory (BPI)***			X	X	X	X	X	X
***Patient Global Impression of Change (PGIC)***			X	X	X	X	X	X
***Adverse events***			X	X	X	X	X	X

### Eligibility

#### Inclusion criteria

For inclusion, patients will be ages 18–65 years, consulting at the pain clinic of the GHdC, with chronic pain (as defined by the IASP) associated with fibromyalgia as defined by the ACR 2016 criteria [4], with a score > 40 on the CSI. They must also be eligible for intravenous ketamine ambulatory treatment according to our latest clinical practice, with no previous experience of treatment with ketamine or S-ketamine for fibromyalgia or other types of chronic pain. All participants will give written informed consent.

#### Exclusion criteria

Pregnant or breastfeeding women (based on patient questioning and urine pregnancy test for women of childbearing age) will be excluded. A pregnancy discovered during the study will be reported to the ethics committee. Women of childbearing age not using an approved contraceptive method with a safety rate > 99% (< 1% on the Pearl index) also will be excluded. Other exclusion criteria include a history of psychiatric disorders (e.g., psychosis), an inability to give informed consent (including because of a linguistic barrier), allergy or contraindication to any of the products used in the trial, and any medical condition or medication inconsistent with the study, based on the judgment of the clinical investigator.

### Definition of outcome measures

These outcomes have been chosen according to the IMMPACT (Initiative on Methods, Measurement, and Pain Assessment in Clinical Trials) recommendations [17, 18, 28].

#### Primary outcome

Our primary outcome is improvement in pain and/or functional status. Improvement will be defined as clinically significant as follows: for pain relief, a 2-point decrease in the Brief Pain Inventory (BPI) pain severity score [29], and for functionality, a one-point decrease on the BPI pain interference scale [28]. We will compare the proportions of patients with clinically significant improvement in each of these metrics between the S-ketamine and placebo groups.

#### Secondary outcomes

Our secondary outcomes are a 50% pain decrease on a numerical rating scale, impact on quality of life as evaluated using the EQ5D-5 L questionnaire, impact on emotional status using the Hospital Anxiety and Depression Scale questionnaire, and the incidence of adverse events solicited and spontaneously reported to the pain clinic staff. We also will assess patient global satisfaction with the treatment, as evaluated using the Patient Global Impression of Change questionnaire.

All questionnaires will be completed before drug administration, and during the follow-up visits together with secondary outcomes assessments (Table 1).

### Blinding

This is a double-blind trial, so the packaging of the placebo (saline) and the investigational drug (S-ketamine) will be identical and provided by the pharmacy. The staff of the pain clinic, independent from the protocol, will prepare and give the treatment, as well as monitor the patient. They will also hand each patient the questionnaires to complete and fill the adverse events questionnaire.

Each patient will be assigned a code, and data will be collected according to this code, so that investigators are unaware of each patient’s allocation. The investigator will collect anonymous data. The person performing the statistical analysis (JF) is independent of the study protocol.

Because of its dysphoric effects, ketamine could be recognized by the patient. We therefore chose to add clonidine for all patients, because it both has analgesic properties and can alleviate these dysphoric effects [11].

Unblinding will be allowed in case of a suspected unattended serious adverse event, if needed to guarantee its resolution and patient care.

### Patient monitoring

All participants will be equipped with standard, non-invasive hemodynamic monitoring including heart rate, blood pressure, and oxygen saturation. The pain clinic staff, including a physician, will be present during the treatment administration. All adverse events will be monitored continuously and until resolution. If any expected adverse event occurs, it will be treated based on the incident management protocol (see additional file 2).

The patient will have access to a medical consult every 2 weeks for monitoring of pain status and medication adjustment as needed. Any standard treatment outside of the protocol that is relative to fibromyalgia (i.e., EULAR guidelines treatments) can be pursued during the study participation and adapted if necessary. The patient’s baseline treatment should remain the same from the beginning to the end of the protocol, as far as possible. The attending physician will evaluate the need to adapt the treatment if necessary for the patient’s wellbeing. All changes in baseline medication, if required, will be monitored and reported in the case report form.

All patients are free to discontinue the protocol at any time (which is part of the informed consent). Other criteria for study discontinuation for a participant will be lack of adherence to the protocol (for example not coming for the treatment) or serious adverse events.

### Missing data

Participants who do not present for one of the three treatment appointments or one of the three follow-up appointments will be excluded from the study and considered as lost to follow-up. To minimize missing data, pain clinic staff will supervise questionnaires’ completion, ensuring they are adequately filled.

In cases of missing data, we will analyze the cause, according to Rubin [30], and will test whether the data are missing completely at random, missing at random, or missing not at random. Multiple imputation (MI) for longitudinal data will be applied.

### Data handling and record keeping

Patient identity will remain strictly confidential (according to legislation related to the protection of personal data) by means of pseudonymization. All data will be identified by a patient code, keeping it anonymous. Only the investigators’ team will have access to the identity code keys.

Participants will fill out paper questionnaires that will be entered into an electronic case report form (password-secured Excel table) held on the GHdC server. The paper case report forms and electronic database will be stored in the anesthesiology department of the GHdC. The locked database will be transferred to the statistician (JF) for analysis.

All original study records and the database will be kept at the GHdC for 20 years or for a longer period when required by national rules. When the EU clinical trials regulation 536/2014 is applicable, a period of 25 years after the end of the study will be required.

Apart from possible external audit, no internal audit has been scheduled.

### Allocation concealment

On the day of the usual medical consult, inclusion and exclusion criteria will be verified and a written informed consent obtained by the physician. Once the patient is included in the trial, the information will be given to the GHdC pharmacy to enter the patient on the randomization list. The pharmacist of the GHdC will create the randomization list using a computer-generated allocation sequence and will be the only source of access to the randomization key. Patients will be randomized in a 2:1 ratio (S-ketamine: control) in blocs of 15 (S-ketamine 10: control 5). The same randomization method will be used for the two studied cohorts.

Only the person responsible for the randomization, independent from the study protocol, will have the randomization key. The randomization list and a copy will be edited, placed in a sealed envelope, and kept at the GHdC pharmacy.

### Sample size estimation

The sample size was calculated based on a cross-sectional sample of 25 patients already treated with ketamine (dose equivalent to 0.2 mg of S-ketamine), magnesium sulfate, and clonidine for whom we compared data before and 2 weeks after treatment. Applying our primary outcomes, we found that 28% (*n* = 7) met the pain relief objective and 56% (*n* = 14) met the functionality objective. Few data were found concerning a placebo effect, which we assessed at 10% under the conditions of our trial (data extrapolated from Noppers et al. [22]).

The selected size of the study population was calculated based on 0.8 (80%) power at an alpha of 0.0125. The alpha is set to 0.0125 because we will compare two primary outcomes twice, at most: the first comparison will be between the usual care+placebo treatment and usual care+low-dose S-ketamine treatment. If this comparison shows non-significant difference between the two groups, the second comparison will be between the usual care+placebo treatment and the usual care+high-dose S-ketamine treatment. Thus, for the primary outcome, a total of four comparisons will be made, so we divided the targeted *p* value (0.05) by this number of comparisons to arrive at a *p* value that allows for rejection of the null hypothesis of no difference observed between the groups. With a minimum clinically relevant difference of 5%, an expected rate of 10% improvement for the usual care+placebo group, and an expected rate of 25% improvement for the usual care+S-ketamine group, 55 patients per group are needed. Considering a drop-out rate of 20%, 70 patients per group will be needed to ensure that data for 55 per group will be available at the end of follow-up.

This number of patients for inclusion is ambitious, but the pain center treats more than 100 patients with fibromyalgia per year. The duration of recruitment is expected to be one year for the first dose cohort and one more year if a second cohort of patients is needed.

**Fig. 3:**
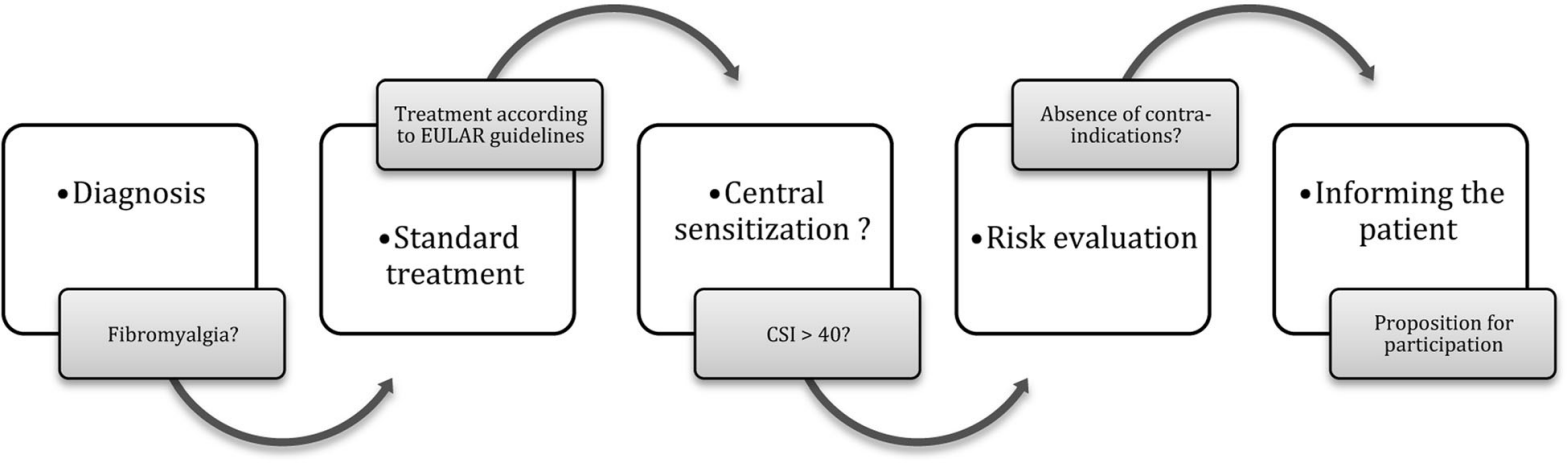
Recruitment process. CSI, Central Sensitization Inventory; EULAR, European League Against Rheumatism

All patients will be recruited by the physicians of the pain clinic during their medical consult, according to the described eligibility criteria (Fig. 3).

### Statistical analysis

An independent and blinded statistician (JF) will perform the statistical analysis. The groups will be compared for the primary and secondary outcomes as well as for group homogeneity. As noted, the statistical significance (*p* value) threshold for both cohorts is set to *p* < 0.0125.

A first analysis will be done after the first cohort follow-up is complete. If the primary outcome is reached, the study will end. However, if the primary outcome is not reached, the study will continue with a second cohort of patients. A complete statistical analysis on both cohorts, if both are conducted, will be performed at the end of the second follow-up. There will not be an interim analysis at any other point.

For discrete data, the Fisher’s exact test will be used, with multiple comparisons among the two or the three groups by means of a logistic regression using the multcomp R package [31]. We will compare continuous data with analysis of variance (ANOVA), testing also for whether the residuals of the ANOVA are normally distributed or not and if the variances are equal between the groups. If the residuals of the ANOVA are not normally distributed or the variances between the groups unequal, we will apply the non-parametric Tukey’s multiple comparisons test using the nparcomp R package [32], and medians and inter-quartile ranges will be reported. The statistical proposition developed by Konietschke [33] and implemented in the nparcomp R Package [32] calculates confidence intervals for rank data. These intervals comprise the zero value when no difference is observed between the groups and do not comprise zero if the groups differ. This mathematical solution thus mimics the behavior of confidence intervals for normally distributed data. When the hypotheses of the ANOVA are met, we will perform Tukey’s multiple comparisons tests for parametric data using the multcomp R package [31] and report results by groups as means ± standard deviations.

To model the evolution of continuous and discrete variables through time, we will use continuous and discrete mixed models [34]. Other effects could be tested, including time, group, time-square, and interactions of group and time or time-square.

As noted, MI will be applied, using the mitml R package. Indeed, maximum likelihood and MI, using all available data in the study, will produce unbiased estimates of the treatment effect and correct *p* values. MI also is a method of choice because it allows for imputation of missing values on the outcome and on the covariate [35] and is valid for the three missingness categories (missing completely at random, missing at random, and missing not at random) [36]. Models obtained via maximum likelihood and MI will be compared. For continuous outcomes, we will look at the residuals of the model, and if these are not normally distributed, we will use the bestNormalize R package to transform the outcome and report the results of this last linear mixed model.

### Ethical considerations

Before initiation, the trial protocol and any documents handed to the patient are reviewed and approved by the independent local ethics committee of the GHdC (rue Marguerite Depasse 6, 6060 Gilly). The protocol also covers the investigator’s responsibility to inform the committee of trial progress (at least once a year), any amendment or modification to the protocol, any serious adverse events, and the end of the trial. In addition to the ethics committee, any serious adverse event will be reported to the AFMPS (Agence fédérale des médicaments et des produits de santé) in the Development Safety Update Report every year. All important protocol modifications will be communicated by the principal investigators to the study coordinator (AG) who will contact relevant parties.

This is a single center academic study for which our ethical committee did not request data monitoring and other committees.

Written informed consent based on voluntary participation has to be obtained before inclusion of each participant, who will be informed of the aims, procedures, and risks related to the trial. At any time during the trial, the participant will have the right to withdraw participation freely without any penalty (Additional file 4).

## Discussion

Fibromyalgia is a primary chronic pain disease that results in progressive deterioration of quality of life, as well as a reduction in relational and functional capacity. Several brain imaging and psychophysical studies support the hypothesis that a central sensitization phenomenon is involved in the etiology of the disease [37, 38]. In 2012, Mayer et al. introduced the CSI as a screening tool to help clinicians in identifying patients with a central sensitization syndrome [39]. Using a receiver operating characteristic analysis in a cohort of 121 patients referred to a multidisciplinary pain center, Neblett et al. determined that a CSI score of 40 out of 100 best distinguished patients with a central sensitization syndrome from those without it [8].

Central sensitization is a condition in which neural “dysregulation” and hyperexcitability lead to a hypersensitivity to nociceptive as well as non-nociceptive stimuli [40]. NMDAR, present in the central nervous system, has been identified as one of the receptors involved in central sensitization [41]. Ketamine exerts its analgesic, psychomimetic, and antidepressant effects through several pathways. One of its main mechanisms of action relies on its non-competitive antagonism of the NMDAR through its phencyclidine binding site, decreasing the frequency of channel opening and duration of time spent in the open (active) state [42]. Several studies have evaluated the effectiveness of ketamine in different chronic pain diseases. In a recent meta-analysis that included seven randomized controlled trials, Orhurhu et al. [14] determined that intravenous ketamine provides a significant short-term analgesic effect in refractory chronic pain. However, the level of evidence was low because all but one included study had a high risk of bias. The authors also noted some evidence of a dose-response relationship. Among the seven studies reviewed, only one concerned patients with fibromyalgia [22]. The authors concluded that additional research is required to determine the ideal patients and conditions for treatment with ketamine, the optimal dosing regimen, and compared to stand-alone treatment, any benefit for physical and psychological functioning from combination therapy with this agent, along with long-term adverse effects.

We designed our trial according to the guidelines for clinical trials in chronic pain, based on the recommendations of the IMMPACT group and the Cochrane Collaboration [17, 18, 28, 43]. Our core outcomes include not only pain relief but also functionality, emotional status, quality of life, patient satisfaction, and adverse events, as both societies recommend. Our protocol was designed taking into account the published guidelines in terms of randomization, allocation concealment, double blinding, and 8–12 weeks of follow-up [43]. Regarding the effect of ketamine on pain, we chose as a primary endpoint a 30% pain reduction (representing 2 points on the BPI pain interference scale), which is cited in the literature as the minimally significant clinical difference [29]. We also decided to report a 50% reduction in pain from baseline as a secondary outcome.

We plan to use S-ketamine because this pure dextrorotatory enantiomer of ketamine is about two to four times more potent than the racemic mixture, enabling lower dosage for equianalgesic effects [12]. It also carries the advantage of producing less psychotropic effect, tiredness, and temporary cognitive impairment than the racemic mixture [13]. Our dose-escalation design has the goal of identifying a minimal efficient dose, if there is one, without losing the benefits relative to adverse events. We decided not to use a dose higher than 0.4 mg/kg of S-ketamine, because according to our experience, these doses become too high to ensure safe administration in outpatient treatment.

Based on our ethical considerations, our protocol provides pain treatment for all patients, with clonidine and magnesium sulfate. Clonidine has well-known analgesic properties in patients with chronic pain [44], and NMDAR is one of the targets of magnesium sulfate [24].

The most important strength of our protocol is the inclusion of function as a primary outcome. Pain relief and functional improvement are not linked in a linear way, and some patients will react to a treatment by pain reduction, whereas others will react with a gain of function. For many patients, gain of function can be more important, on a personal as well as a societal level. We tried to build a protocol that reflects our multimodal perception of pain [45].

## Trial status

Protocol version: 2.2 issued on September 30, 2020

Recruitment is expected to start early 2021 and to be completed in about 2 years.

## Supplementary Information

**Additional file 1.** SPIRIT checklist.

**Additional file 2.** Incident management protocol.

**Additional file 3.** WHO trial registration data set.

**Additional file 4.** Informed consent form.

## Data Availability

The dataset used and/or analyzed during the present study will be available from the corresponding author on reasonable request. Results will be communicated and published in a peer-reviewed journal.

## Abbreviations

ACR: American College of Rheumatology
AFMPS: Agence fédérale des médicaments et des produits de santé
ANOVA: analysis of variance
BPI: Brief Pain Inventory
CSI: Central Sensitization Inventory
GHdC: Grand Hôpital de Charleroi
IASP: International Association for the Study of Pain
IMMPACT: Initiative on Methods, Measurement, and Pain Assessment in Clinical Trials
MI: Multiple imputation
NMDAR: N-methyl-D-aspartate receptor
SPIRIT: Standard Protocol Items—Recommendations for Interventional Trials
