# Supplementary material for: The place of S-ketamine in fibromyalgia treatment (ESKEFIB): study protocol for a prospective, single-center, double-blind, randomized, parallel-group, dose-escalation controlled trial
Source: Trials. 2021 Nov 27;22:853. doi: 10.1186/s13063-021-05814-4 (PMC8627027; doi:10.1186/s13063-021-05814-4)
Supplement: Supplementary file 2 — Additional file 2. Incident management protocol. [file 13063_2021_5814_MOESM2_ESM.docx]

Additional File 2: Incident management protocol

| Incident | Treatment |
| --- | --- |
| Nausea/Vomiting | - Alizapride 50 mg slow IV - Dompéridone 10 mg p.o |
| Symptomatic hypotension | 1. Trendelenburg  2. NaCl 0,9% 250 ml  3. Ephedrine titrated by 3-6 mg IV boluses |
| Bradycardia | - Atropine 0,5 mg IV |
| Headache | - Dafalgan 1g - Ibuprofen 400 mg |

IV: Intravenous ; p.o : per os
