# Supplementary material for: The place of S-ketamine in fibromyalgia treatment (ESKEFIB): study protocol for a prospective, single-center, double-blind, randomized, parallel-group, dose-escalation controlled trial
Source: Trials. 2021 Nov 27;22:853. doi: 10.1186/s13063-021-05814-4 (PMC8627027; doi:10.1186/s13063-021-05814-4)
Supplement: Supplementary file 3 — Additional file 3. WHO trial registration data set. [file 13063_2021_5814_MOESM3_ESM.docx]

Additional file 3: WHO trial registration data set:

| Data category | Information |
| --- | --- |
| Primary Registry and Trial Identifying Number | ClinicalTrials.gov NCT04436250 |
| Date of Registration in Primary Registry | June 18, 2020 |
| Secondary Identifying Numbers | EudraCT 2020-000473-25  GHdC reference : G1-2020-E020 |
| Source(s) of Monetary or Material Support | Grand Hôpital de Charleroi (GHdC) |
| Primary Sponsor | Grand Hôpital de Charleroi |
| Secondary Sponsor(s) | None |
| Contact for Public Queries | Zuzana Javorcikova (z.javorcikova.pro@gmail.com) |
| Contact for Scientific Queries | Zuzana Javorcikova (z.javorcikova.pro@gmail.com) |
| Public Title | S-ketamine for fibromyalgia treatment |
| Scientific Title | ESKETamine for FIBromyalgia treatment |
| Countries of Recruitment | Belgium |
| Health Condition(s) or Problem(s) Studied | Fibromyalgia |
| Intervention(s) | Active comparator: S-ketamine (dose 0,2 mg/kg and 0,4 mg/kg)  Placebo: NaCl 0,9%  Non-Investigational Medicinal Products:  - Clonidine 1 mcg/kg  - Magnesium sulphate 40 mg/kg |
| Key Inclusion and Exclusion Criteria | Age eligibility for the study 18-65 years; sexes : both ; Accepts healthy volunteers : no  Inclusion criteria: fibromyalgia, score > 40 on the central sensitization inventory  Exclusion criteria: Allergy or contraindication to any of the products used in the trial, pregnancy, breastfeeding, psychiatric disorders such as psychosis, incapacity of giving an informed consent |
| Study Type | Interventional, double blinded, parallel group, randomized study  Primary purpose: treatment  Phase III |
| Date of First Enrollment | To be started December 2020 |
| Target Sample Size | 210 |
| Recruitment Status | Pending |
| Primary Outcome(s) | Improvement in patient’s pain and/or function status (evaluated by the Brief Pain Inventory) |
| Key Secondary Outcomes | 50% pain decrease, impact on quality of life, impact on emotional status, incidence of adverse events, patient global satisfaction |
| Ethics Review | Approved on October 10, 2020 by the GHdC ethics committee (comite.ethique@ghdc.be) |
| Expected completion date | August 2022 |
