# Supplementary material for: The place of S-ketamine in fibromyalgia treatment (ESKEFIB): study protocol for a prospective, single-center, double-blind, randomized, parallel-group, dose-escalation controlled trial
Source: Trials. 2021 Nov 27;22:853. doi: 10.1186/s13063-021-05814-4 (PMC8627027; doi:10.1186/s13063-021-05814-4)
Supplement: Supplementary file 4 — Additional file 4. Informed consent form. [file 13063_2021_5814_MOESM4_ESM.docx]

Titre de l’étude : Perfusions intraveineuses de S-kétamine dans les syndromes fibromyalgiques - ESKEFIB

Promoteur de l’étude : Grand Hôpital de Charleroi ; Rue Marguerite Depasse, 6 ; B-6060 Charleroi ; Belgique

Comité d’Ethique Médicale *:* Comité d’éthique du Grand Hôpital de Charleroi, Rue Marguerite Depasse, 6 | 6060 Gilly. ([comite.ethique@ghdc.be](mailto:comite.ethique@ghdc.be) ; tel. 071/10.52.56)

Médecins investigateurs locaux :

- Dr Jean-Paul Lechat, chef de service d’anesthésie, Site Notre-Dame, Grand Rue 3 ; B-6000 Charleroi ; Belgique. (jeanpaul.lechat@ghdc.be ; tel : 071/10 40 71)

- Dr Philippe Van Der Linden, service d’anesthésie, Site Site Notre-Dame, Grand Rue 3 ; B-6000 Charleroi ; Belgique. ([Philippe.VANDERLINDEN@ghdc.be](mailto:Philippe.VANDERLINDEN@ghdc.be) )

- Dr Michel Dangoisse, service d’anesthésie, Site Site Notre-Dame, Grand Rue 3 ; B-6000 Charleroi ; Belgique. ([Michel.dangoisse@ghdc.be](mailto:Michel.dangoisse@ghdc.be%20) )

- Dr Stephane.Nikis, service d’anesthésie, Site Sainte-Thérèse, Rue Trieu Kaisin 134 ; 6061 Charleroi ; Belgique ([Stephane.Nikis@ghdc.be](mailto:Stephane.Nikis@ghdc.be))

- Dr Zuzana Javorcikova, assistante dans le service d’anesthésie, Site Site Notre-Dame, Grand Rue 3 ; B-6000 Charleroi ; Belgique. (z.javorcikova.pro@gmail.com)

**I Information essentielle à votre décision de participer**

**Introduction**

Vous êtes invité à participer à une étude clinique destinée à évaluer un médicament connu mais expérimental dans le cadre du traitement de votre maladie, la fibromyalgie.  Un médicament expérimental est un médicament faisant encore l'objet d'études pour évaluer son efficacité, sa sécurité d'emploi ou son mécanisme d'action.

Le promoteur et le médecin investigateur espèrent que ce médicament peut présenter des avantages pour le traitement de patients atteints de la même maladie que la vôtre. Néanmoins, il n’y a aucune garantie que vous tirerez un bénéfice de votre participation à cette étude.

Avant que vous n’acceptiez d’y participer, nous vous invitons à prendre connaissance de ses implications en termes d’organisation, avantages et risques éventuels, afin que vous puissiez prendre une décision en toute connaissance de cause. Ceci  s’appelle donner un « consentement éclairé ».

Veuillez lire attentivement ces quelques pages d’information et poser toutes les questions que vous souhaitez à l’investigateur ou à la personne qui le représente. Ce document comprend 3 parties : l’information essentielle à votre prise de décision, votre consentement écrit et des informations complémentaires (annexes) qui détaillent certaines parties de l’information de base.

**Si vous participez à cette étude clinique, vous devez savoir que :**

- Cette étude clinique est mise en œuvre après évaluation par un comité d’éthique.
- Votre participation est volontaire et doit rester libre de toute contrainte. Elle nécessite la signature d’un document exprimant votre consentement.  Même après l’avoir signé, vous pouvez arrêter de participer en informant le médecin investigateur. Votre décision de ne pas ou de ne plus participer à l’étude n’aura aucun impact sur la qualité de vos soins ni sur vos relations avec le médecin investigateur.
- Les données recueillies à cette occasion sont  confidentielles et votre anonymat est garanti lors de la publication des résultats.
- Une assurance a été souscrite au cas où vous subiriez un dommage lié à votre participation à cette étude clinique.
- Aucun frais ne vous sera facturé pour les visites / consultations, examens ou traitements spécifiques à cette étude.
- Vous pouvez toujours contacter le médecin investigateur ou un membre de son équipe si vous avez besoin d’informations complémentaires.

Un complément d’informations sur vos « Droits de participant à une étude clinique » est fourni en annexe III. 3.

**Objectifs et description du protocole de l’étude**

Nous vous proposons de participer à une étude clinique portant sur la S-kétamine qui devrait inclure environ 210 patients dont tous résident en Belgique.

L’objectif de l’étude est d’évaluer l’effet de l’adjonction de la S-kétamine à un mélange de produits antalgiques au niveau de l’amélioration de vos douleurs et votre qualité de vie.

Cette étude vous est proposée car vous répondez aux critères suivants:

- Diagnostic de fibromyalgie d’après les derniers critères de l’ACR^[[1]](#footnote-1)^ (2016)

- Age entre 18-65 ans

- Vous n’avez pas bénéficié de perfusion de kétamine pour les douleurs chroniques auparavant

-  Selon le questionnaire que vous avez rempli (Inventaire de Sensibilisation Centrale) vous présentez un profil de la maladie dans lequel ce type de traitement pourrait être efficace.

Il s’agit d’une étude randomisée en double aveugle comparant la S-kétamine (le médicament testé), à un placebo “actif”, c’est à dire un mélange de produits à activité antalgique reconnue autres que la S-kétamine. En « double aveugle » signifie que vous n’allez pas savoir si vous recevez la S-kétamine ou pas en plus des autres traitements antalgiques, et le médecin ne le saura pas non plus. Ces informations ne seront révélées qu’une fois l’étude terminée ou en cas de nécessité médicale. Le terme « randomisée » signifie que le hasard déterminera si vous recevrez de la S-kétamine ou non.

Dans le cadre de notre étude, vous aurez 2 chances sur 3 de recevoir la molécule.

Il y aura une première cohorte de patients qui recevra une dose de 0,2 mg/kg de S-kétamine. Un second groupe pourra éventuellement recevoir une dose de 0,4 mg/kg de S-kétamine après analyse des données du premier groupe.

**Déroulement de l’étude**

Votre participation à l’étude durera environ 12 semaines et impliquera 6 visites à deux semaines d’intervalle entre chacune.

De même quelques procédures ou examens supplémentaires seront requis dans le cadre de l’étude (voir détails annexe III.1)

Votre participation à l’étude s’inscrivant dans le cadre de la prise en charge de votre situation clinique, une partie des visites et examens que nous allons décrire fait partie de la norme de soin en usage dans notre hôpital tandis que d’autres sont proposées par l’étude.

L’étude se déroule exclusivement à la clinique de la douleur du GHdC (Site Sainte-Thérèse, Rue Trieu Kaisin 134, 6061 Charleroi). La participation vous est proposée par le médecin algologue que vous voyez en consultation et qui pose le diagnostic de fibromyalgie une fois que vous avez fait les tests nécessaires. La première phase de l’étude se déroule en trois visites espacées de deux semaines pendant lesquelles vous recevrez le traitement. La deuxième phase de l’étude se déroulera lors de plusieurs consultations (3) de suivi. Lors de chaque visite, il vous sera demandé de remplir divers questionnaires permettant d’évaluer l’évolution des symptômes de votre maladie au niveau de votre douleur et votre qualité de vie.

Votre traitement consiste en l’administration d’une perfusion en hospitalisation de jour. Vous recevrez un traitement antalgique comportant ou non de la S-kétamine. L'hospitalisation de jour se fait à la clinique de la douleur et la durée de la perfusion sera de trois heures. Un médecin sera toujours disponible et l’équipe infirmière sera présente pour votre surveillance.

La S-kétamine, qui est le médicament étudié, agit en tant qu’antalgique au niveau du système nerveux central. Vous pouvez vous trouver dans un groupe ne recevant pas de S-kétamine, un groupe recevant la dose de 0,2 mg/kg de S-kétamine ou un groupe recevant 0,4 mg/kg de S-kétamine. Peu importe le groupe dans lequel vous vous trouvez, d’autres antidouleurs vous seront administrés dans la perfusion.

Cette pratique s’inscrit dans notre pratique clinique habituelle. La seule différence consiste en la fréquence des rendez-vous, qui dans le cadre de l’étude est plus rapprochée afin d’avoir un suivi régulier.

Si vous acceptez de participer à l’étude et si vous répondez à toutes les conditions requises pour être enrôlé(e) dans l’étude, vous passerez les tests et examens décrits ci-dessous :
- Un électrocardiogramme (si vous n’en avez pas eu dans l’année précédant votre inclusion à l’étude)

- Un test de grossesse si vous êtes en âge de procréer

Sachez que si pour quelque raison que ce soit vous devez quitter l’étude, vous pouvez le faire à n’importe quelle étape de celle-ci.

**Risques et inconvénients**

**A : Interactions médicamenteuses ou autres**

La S-kétamine est contre-indiquée dans les cas suivants (cfr. notice du médicament) :

- Hypersensibilité (allergie) à la substance active ou à l’un des excipients

- Lorsqu’une augmentation de la pression artérielle constitue un risque grave.

- Troubles cardiovasculaires graves.

- Augmentation de la pression du liquide cérébrospinal et affections intracrâniennes sévères.

Des interactions médicamenteuses ont été observées avec les médicaments suivants : l’ergométrine, la théophylline et l’aminophylline.

L’usage pendant la grossesse n’est pas recommandé.

Il est préférable de ne pas conduire après l’administration du traitement, il est donc nécessaire de rentrer accompagné(e).

Ces contre-indications et interactions seront prises en compte par votre médecin avant de vous proposer de participer à l’étude.

**B : Effets secondaires du médicament étudié**

Tous les médicaments ont des effets secondaires connus ou imprévisibles.  Si les études précédentes ont montré que  l’association de médicaments de cette étude était habituellement bien toléré(e), vous pourriez cependant éprouver les effets secondaires suivants :

- Très fréquents: /

- Fréquents: Réactions de réveil telles que rêves agités, y compris cauchemars, vertiges et agitation motrice, vision trouble, augmentation temporaire de la fréquence cardiaque (tachycardie temporaire), augmentation de la tension artérielle et des battements du cœur (une augmentation d’environ 20% du niveau de départ est fréquente), augmentation de la résistance vasculaire dans la circulation pulmonaire, et augmentation de la sécrétion mucosale, augmentation de la consommation d’oxygène, laryngospasmes et dépression respiratoire temporaire (le risque de dépression respiratoire dépend en général de la dose et de la vitesse d’injection), nausées et vomissements, salivation augmentée,

D’autres risques et inconvénients inconnus à ce jour peuvent éventuellement apparaitre.  Il est donc très important de signaler rapidement tout nouveau problème de santé au médecin en charge de votre suivi, que vous pensiez ou non qu’il soit en rapport avec l’étude.

Une liste exhaustive des effets secondaires de chacun des médicaments faisant partie du traitement peut être retrouvée en annexe (Annexe III. 2.)

**C : Contraception, grossesse et allaitement**

Participant féminin : Etant donné que les effets de la S-kétamine sur un enfant à naître ou un nourrisson ne sont pas parfaitement connus, vous ne serez pas autorisée à participer à cette étude clinique si vous êtes enceinte, souhaitez être enceinte ou si vous allaitez.

Si vous choisissez de participer à cette étude, vous devrez utiliser l’une des méthodes contraceptives autorisées (de manière à ce que vous ne tombiez pas enceinte). Votre médecin discutera avec vous les différentes options appropriées qui sont présentées en annexe (Annexe III.2).

Participant masculin : Il n’y a pas de données disponibles des effets potentiels de la S-kétamine sur la fertilité mâle ou femelle. Il n’y a pas à ce jour de preuves montrant des risques pour votre partenaire si vous bénéficiez d’un traitement par la S-kétamine. Par principe de précaution, une protection contraceptive est toute fois préconisée pour la durée de l’étude. Vous vous engagez à informer votre partenaire de votre participation à cette étude et du risque potentiel pour un embryon ou un fœtus.

**D : Risques liés aux procédures d’évaluation propres à l’étude**

L’évaluation du traitement se fera sous forme de questionnaires. Il vous faudra répondre de manière complète et la plus honnête possible, même si la longueur des questionnaires peut vous paraître importante. La réponse aux questionnaires ne comporte pour vous aucun risque particulier, mais aidera grandement le médecin à évaluer l’effet du traitement sur votre douleur et votre qualité de vie.

**Notification d’informations nouvelles**

Il se peut que pendant le déroulement d’une étude clinique, de nouvelles informations importantes sur la S-kétamine deviennent disponibles. Vous serez informé(e) de tout élément nouveau susceptible d’affecter votre décision de poursuivre votre participation à cette étude.

Dans ce cas, on vous demandera de signer soit un complément au formulaire de consentement, soit un nouveau document d’information et de consentement. Si, à la lumière de la nouvelle information, vous décidez de mettre un terme à votre participation à l’étude, votre médecin investigateur veillera à ce que vous continuiez d’être traité(e) de la meilleure façon qui soit.

**Bénéfices**

Si vous acceptez de participer à cette étude, la S-kétamine pourra ou non s’avérer bénéfique pour le traitement de la fibromyalgie ou diminuer vos symptômes.

Les informations obtenues grâce à cette étude peuvent contribuer à une meilleure connaissance de l’utilisation de ce médicament dans le cadre de la prise en charge de la fibromyalgie chez de futurs patients.

**Traitement alternatif :**

D’autres traitements existent pour votre affection. Probablement vous en aviez déjà bénéficié auparavant. Certains anti-épileptiques, certains anti-dépresseurs, la kinésithérapie et la prise en charge dans un centre de la douleur en font partie. La S-kétamine en est également un. Elle ne remplace pas un traitement qui fait partie de votre traitement habituel, il s’agit d’un traitement additionnel.

Votre médecin discutera avec vous de ces traitements.

**Retrait de l’étude**

Votre participation est volontaire et vous avez le droit de vous retirer de l’étude pour quelque raison que ce soit, sans devoir vous justifier.  Néanmoins, il peut être utile pour le médecin investigateur et pour le promoteur de l’étude de savoir si vous vous retirez parce que les contraintes du traitement sont trop importantes (trop d’effets secondaires désagréables par exemple).

Il est aussi possible que ce soit le médecin investigateur qui vous retire de l’étude parce que vous êtes enceinte, parce qu’il pense que c’est le mieux pour votre santé ou qu’il constate que vous ne respectez pas les consignes données aux participants.

Enfin, il arrive parfois que les autorités compétentes nationales ou internationales, le comité d’éthique qui a initialement approuvé l’étude ou le promoteur interrompent l’étude parce que les informations recueillies montrent que le traitement étudié n’est pas efficace (n’apporte pas assez d’amélioration de la santé des participants), que le traitement étudié occasionne plus d’effets secondaires ou des effets secondaires plus graves que prévu ou pour toute autre raison comme par exemple la décision d’arrêter les recherches et le développement du médicament étudié.

**Traitement après l’arrêt de l’étude**

Dans toutes ces situations de retrait de l’étude mais également lorsque le temps de participation prévu est terminé, votre médecin évaluera votre état de santé et vous prescrira le meilleur traitement disponible.

Au terme de l’étude votre médecin jugera de l’intérêt de poursuivre ce traitement et de recevoir le médicament étudié pendant une nouvelle période. Votre médecin vous fera cette proposition s’il estime que cette option vous est favorable.

**Si vous participez à cette étude clinique, nous vous demandons :**

1. De collaborer pleinement au bon déroulement de cette recherche.
2. De ne masquer aucune information relative à votre état de santé, aux médicaments que vous prenez ou aux symptômes que vous ressentez.
3. De ne participer à aucune autre recherche clinique concernant un traitement expérimental, qu’il s’agisse d’un médicament, d’un dispositif médical ou d’une procédure, tant que vous participerez à la présente étude.

**Vous devez également savoir que :**

- pour votre sécurité, il est souhaitable que votre médecin généraliste, si vous en avez un, ou d'autres médecins spécialistes en charge de votre santé soient informés de votre participation à cette étude. Nous vous demanderons de confirmer votre accord mais respecterons votre éventuelle volonté de ne pas les informer.

**Contact**

Si vous avez besoin d’informations complémentaires, mais aussi en cas de problème ou d’inquiétude, vous pouvez contacter le médecin investigateur Michel Dangoisse ou un membre de son équipe de recherche au numéro de téléphone suivant 071/109634.

En cas d'urgence, vous pouvez contacter un médecin au numéro de téléphone suivant 071/104097.

En dehors des heures de consultation, adressez-vous aux urgences de votre hôpital en leur signalant que vous participez à une étude clinique.  Votre dossier contiendra les informations utiles au médecin de garde concernant cette étude clinique.

Si vous avez des questions relatives à vos droits de participant à une étude clinique, vous pouvez contacter le médiateur des droits du patient de votre institution via le numéro de téléphone: 071/10 52 13 ou via notre officier de sécurité dpo@ghdc.be. Si nécessaire, ce dernier peut vous mettre en contact avec le comité d’éthique.

Titre de l’étude : Perfusions intraveineuses d’S-kétamine dans les syndromes fibromyalgiques

**II Consentement éclairé**

**Participant**

Je déclare que j’ai été informé sur la nature de l’étude, son but, sa durée, les éventuels bénéfices et risques et ce que l’on attend de moi.  J’ai pris connaissance du document d’information et des annexes à ce document.

J’ai eu suffisamment de temps pour y réfléchir et en parler avec une personne de mon choix comme mon médecin généraliste ou un membre de ma famille.

J’ai eu l’occasion de poser toutes les questions qui me sont venues à l’esprit et j’ai obtenu une réponse satisfaisante à mes questions.

J’ai compris que ma participation à cette étude est volontaire et que  je suis libre de mettre fin à ma participation à cette étude sans que cela ne modifie mes relations avec l’équipe thérapeutique en charge de ma santé.

J’ai compris que des données me concernant seront récoltées pendant toute ma participation à cette étude et que le médecin investigateur et le promoteur de l’étude se portent garant de la confidentialité de ces données.

Je consens au traitement de mes données personnelles selon les modalités décrites dans la rubrique traitant de garanties de confidentialité (annexe III.3). Je donne également mon accord au transfert et au traitement de ces données dans d’autres pays que la Belgique.

J’accepte que les données de recherche récoltées pour les objectifs de la présente étude puissent être traitées ultérieurement pour autant que ce traitement soit limité au contexte de la présente étude pour une meilleure connaissance de la maladie et de son traitement.

J’accepte / n’accepte pas (biffer la mention inutile) que mon médecin généraliste ou d'autres médecins spécialistes en charge de ma santé soient informés de ma participation à cette étude clinique.

J’ai reçu une copie de l’information au participant et du consentement éclairé.

Nom : ________________________Prénom :______________________

Date :_________________________Signature :____________________

du patient

**Médecin Investigateur**

Je soussigné, ………………………………………………………………….médecin investigateur confirme avoir fourni oralement les informations nécessaires sur l'étude et avoir fourni un exemplaire du document d’information au participant.

Je confirme qu'aucune pression n'a été exercée pour que le patient accepte de participer à l'étude et que je suis prêt à répondre à toutes les questions supplémentaires, le cas échéant.

Je confirme travailler en accord avec les principes éthiques énoncés dans la dernière version de la « Déclaration d’Helsinki », des « Bonnes pratiques Cliniques » et de la loi belge du 7 mai 2004, relative aux expérimentations sur la personne humaine.

Nom : ____________________Prénom : _____________________________

Date :_____________________Signature : _________________________

du médecin investigateur

Titre de l’étude : Perfusions intraveineuses d’S-kétamine dans les syndromes fibromyalgiques

**III Informations complémentaires**

**1 : Compléments d’informations sur l’organisation de l’étude**

Tableau récapitulatif de vos visites :

|  | **Moment** | **Ce qu’on va faire** | **Ce que vous allez faire** |
| --- | --- | --- | --- |
| **Recrutement** | ***Consultation chez votre médecin algologue*** | - Explication de l’étude  - Vous faire passer un test de diagnostic de la fibromyalgie et un questionnaire mettant ou non en évidence le phénomène de sensibilisation centrale  - Prescription de l’ ECG^[[2]](#footnote-2)^  - Prescription d’un test de grossesse  - Prise de vos rendez-vous futurs | - Marquer votre consentement  - Aller faire un électrocardiogramme  - Faire un test de grossesse si vous êtes en âge d’avoir des enfants  - Vous munir d’une méthode de contraception reconnue si vous être en âge d’avoir des enfants |
| **Phase de traitement** | **1^e^ séance de traitement** | Administration du traitement antalgique en hospitalisation de jour | - Remplir les questionnaires suivants : CSI, BPI, EQ5D-5L, HADS*  - Signaler les effets secondaires éventuels |
|  | **2^e^ séance de traitement** | Administration du traitement antalgique en hospitalisation de jour | - Remplir les questionnaires suivants : BPI, PGIC*  - Signaler les effets secondaires éventuels |
|  | **3^e^ séance de traitement** | Administration du traitement antalgique en hospitalisation de jour | - Remplir les questionnaires suivants : BPI, PGIC*  - Signaler les effets secondaires éventuels |
| **Phase de suivi** | **1^e^ consultation de suivi** | Consultation | - Remplir les questionnaires suivants : BPI, PGIC*  - Signaler les effets secondaires éventuels |
|  | **2^e^ consultation de suivi** | Consultation | - Remplir les questionnaires suivants : BPI, PGIC*  - Signaler les effets secondaires éventuels |
|  | ***3^e^ consultation de suivi*** | Consultation | - Remplir les questionnaires suivants : CSI, BPI, EQ5D-5L, HADS*  - Signaler les effets secondaires éventuels |

A partir de la phase de traitement, chaque visite sera espacée d’une période de deux semaines. Seules les consultations en *italique* sur fond gris sont à charge du patient car elles rentrent dans le cadre des consultations de routine.

* Il s’agit de questionnaires permettant d’évaluer votre douleur et votre qualité de vie :

CSI : Inventaire de sensibilisation centrale - BPI : Brief Pain Inventory - EQ5D-5L : questionnaire européen d’évaluation de la qualité de vie - HADS : Hospital Anxiety and Depression Scale - PGIC : Patient Global Impression of Change

**2 : Complément d’informations sur les risques liés à la participation à l’étude**

Détails sur les effets secondaires des différents médicaments selon leur fréquence d’apparition :

S-kétamine: produit étudié

| **Très fréquent**  (Plus d’un patient sur 10) | / |
| --- | --- |
| **Fréquent**  (Plus d’un patient sur 100 mais moins d’un patient sur 10) | - Réactions de réveil après l’anesthésie. Ces réactions comprennent rêves agités, cauchemars, vertiges et agitation  - Vision trouble  - Augmentation transitoire de la fréquence cardiaque, augmentation de la tension artérielle  - Effets sur la respiration pendant l’anesthésie  - Nausées et vomissements, salivation augmentée |
| **Peu fréquent**  (Plus d’un patient sur 1000 mais moins d’un patient sur 100) | - Augmentation des mouvements du corps tels que contractions musculaires, ressemblant parfois à des convulsions, et augmentation des mouvements oculaires  - Vision double, augmentation de la pression dans l’œil  - Éruption cutanée  - Douleur et/ou rougeur au site d’injection |
| **Rare**  (Plus d’un patient sur 10.000 mais moins d’un patient sur 1000) | - Réactions allergiques graves  - Rythme cardiaque irrégulier ou ralenti  - Tension artérielle basse |
| **Fréquence non rapportée** | - Hallucinations, dysphorie, anxiété et désorientation  - Résultats anormaux du test de la fonction du foie  - Lésion du foie |

Clonidine : antalgique utilisé dans le traitement

| **Très fréquent**  (Plus d’un patient sur 10) | étourdissements, sédation, sécheresse de la bouche, chute de la tension artérielle lors du passage en position debout |
| --- | --- |
| **Fréquent**  (Plus d’un patient sur 100 mais moins d’un patient sur 10) | céphalées, fatigue,  constipation, nausées, vomissement,  douleur de la glande salivaire,  troubles érectiles,  dépression, troubles du sommeil |
| **Peu fréquent**  (Plus d’un patient sur 1000 mais moins d’un patient sur 100) | rythme cardiaque lent, sensation cutanée anormale (paresthésie), prurit, rash, urticaire,  syndrome de Raynaud (troubles de la circulation sanguine dans les pieds et les mains, causant par exemple une décoloration des doigts), malaise, cauchemars, hallucinations, troubles de la perception |
| **Rare**  (Plus d’un patient sur 10.000 mais moins d’un patient sur 1000) | troubles de la conduction nerveuse du cœur,  diminution du flux des larmes, symptômes d’une obstruction des intestins, perte de cheveux,  augmentation de volume des glandes mammaires chez l’homme, augmentation transitoire du taux sanguin du sucre glucose |
| **Fréquence indéterminée** | rythme cardiaque lent et irrégulier, troubles de l’acuité visuelle causés par des troubles de l’adaptation du diamètre de la pupille, confusion,  diminution de la libido |

Sulfate de magnésium : antalgique utilisé dans le traitement

La fréquence des effets secondaires n’a pas été établie, mais les effets suivants ont été pu être rapportés :

- Prolongation du temps de saignement et inhibition de l’agrégation des plaquettes du sang

- Dilatation de ces vaisseaux, bouffées vasomotrices (= pouvant provoquer une modification du calibre des vaisseaux), modifications électrocardiographiques, ralentissement des battements du cœur, baisse de tension, défaillance de la circulation du sang, défaillance du cœur, voire arrêt du cœur

- Diminution de la libération de neurotransmetteurs avec comme conséquences : blocage de la transmission neuromusculaire, forte diminution des réflexes, paralysie flasque, confusion et dépression du système nerveux central

- Transpiration, chute de la température du corps, diminution de la quantité de calcium dans le sang, diminution de la quantité de phosphate dans le sang, excès de potassium dans le sang et épaississement du sang

- Nausées et vomissements, qui sont les signes précoces d’hypermagnésémie ; occlusion de l’intestin par paralysie. L’occlusion de l’intestin par paralysie est une complication qui survient rarement lorsque le sulfate de magnésium est utilisé comme agent tocolytique. Une occlusion intestinale a

également été rapportée chez le nouveau-né suite à l’administration de sulfate de magnésium comme tocolytique.

- Insuffisance rénale. L’excès de magnésium dans le sang peut contribuer au développement d’une mauvaise fonction des reins chez les patients sans antécédents de dysfonctionnement rénal. Par ailleurs, les patients avec une mauvaise fonction des reins pré-existante sont prédisposés à développer un excès de magnésium dans le sang, étant donné la diminution de l’élimination rénale.

- Vision trouble, crainte de la lumière, vision double, diminution de la vue et secousses rythmiques des yeux

- Souffle court, dépression respiratoire et œdème pulmonaire. Il peut également survenir une paralysie respiratoire qui peut être fatale

- Rash

- Faiblesse musculaire excessive.

- Maux de tête, dysarthrie (= difficulté d'élocution, de parole).

**Contraception  – participante enceinte / partenaire d’un participant enceinte / grossesse - préjudice pour l'enfant à naître**

Participant féminin :

La sécurité d'emploi de la S-kétamine pendant la grossesse n'a pas été établie et son emploi n’est pas recommandé pendant la grossesse. L’S-kétamine est excrétée dans le lait maternel, mais aux doses thérapeutiques, un effet chez l’enfant semble improbable. Il est cependant recommandé dans le cadre de cette étude de ne pas allaiter pendant une période suffisamment longue de façon à permettre l'élimination complète du produit.

Une précaution par méthode de contraception reconnue (dont les taux d’échec sont inférieurs à 1 % car aucune méthode de contraception n’est efficace à 100%) doit être prise et utilisée correctement.

1. Implant contraceptif
2. Vasectomie
3. Dispositif intra-utérin au lévonogestrel ou en cuivre
4. Stérilisation féminine
5. Pilule oestroprogestative ou progestative
6. Injectable progestatif et injectables mensuels
7. Patch contraceptif
8. Anneau vaginal

Participant masculin :

Il n’y a pas de données disponibles des effets potentiels de la S-kétamine sur la fertilité mâle ou femelle. Le besoin formel d’une contraception n’a pas été établi chez les sujets masculins traités par S-kétamine. Des mesures de protection sont toutefois conseillées par principe de précaution.

Si toutefois une grossesse débute chez vous ou votre partenaire dans le décours de l’étude, veuillez en informer votre médecin.

**Risques associées aux procédures de l’étude clinique**

Un électrocardiogramme est une procédure rapide et indolore.

Un test de grossesse peut être effectué par analyse urinaire, et est également indolore.

**3 : Complément d’informations sur la protection et les droits du participant à une étude clinique**

***Comité d'Ethique***

Cette étude a été évaluée par un Comité d'Ethique indépendant, à savoir le Comité d'Ethique (Comité d’éthique du Grand Hôpital de Charleroi), qui a émis un avis favorable.  Les Comités d'Ethique ont pour tâche de protéger les personnes qui participent à un essai clinique. Ils s'assurent que vos droits en tant que patient et en tant que participant à une étude clinique sont respectés, qu'au vu des connaissances actuelles, la balance entre risques et bénéfices reste favorable aux participants, que l'étude est scientifiquement pertinente et éthique.
En aucun cas vous ne devez prendre l'avis favorable du Comité d'Ethique comme une incitation à participer à cette étude.

***Participation volontaire***

Avant de signer, n’hésitez pas à poser toutes les questions que vous jugez utiles.  Prenez le temps d’en parler à une personne de confiance si vous le souhaitez.

Votre participation à l’étude est volontaire et doit rester libre de toute contrainte: ceci signifie que vous avez le droit de ne pas y participer ou de vous retirer sans justification même si vous aviez accepté préalablement d’y participer.  Votre décision ne modifiera en rien vos relations avec le médecin investigateur et la qualité de votre prise en charge thérapeutique future.

Toutefois, il est conseillé pour votre sécurité, de prévenir le médecin investigateur si vous avez décidé d’arrêter votre participation à l’étude.

Si vous acceptez  d’y participer, vous signerez le formulaire de consentement éclairé.  Le médecin investigateur signera également ce formulaire et confirmera ainsi qu'il vous a fourni les informations nécessaires sur l'étude.  Vous recevrez l’exemplaire qui vous est destiné.

***Coûts associés à votre participation***

Le promoteur a prévu de dédommager l’hôpital pour le temps consacré à l’étude par le médecin investigateur et son équipe, pour les consultations spécifiques à l’étude et pour tous les examens programmés dans le cadre de cette étude.  De même le traitement étudié est à charge du promoteur.

Si vous décidez de participer à cette étude, ceci n’entraînera donc pas de frais supplémentaires pour vous ou votre organisme assureur. Les visites et procédures identifiées comme propres à l’étude dans le descriptif du déroulement de l’étude dans le tableau de la page 7 sont à charge du promoteur. Seuls les frais correspondant aux prestations médicales de pratique courante dans votre situation clinique, peuvent vous être facturés.

***Garantie de confidentialité***

Votre participation à l’étude signifie que vous acceptez que le médecin investigateur recueille des données vous concernant et que le promoteur de l’étude les utilise dans un objectif de recherche et dans le cadre de publications scientifiques et médicales.

Vous avez le  droit de demander au médecin investigateur quelles sont les données collectées à votre sujet et quelle est leur utilité dans le cadre de l'étude.  Ces données concernent votre situation clinique actuelle mais aussi certains de vos antécédents, les résultats des examens réalisés dans le cadre d’une prise en charge selon les standards actuels de votre santé et bien entendu les résultats des examens requis par le protocole. Vous disposez d’un droit de regard sur ces données et le droit d’y apporter des rectifications au cas où elles seraient incorrectes (droits garantis par la loi du 22 août 2002 relative aux droit du patient, par la loi du 30 juillet 2018 relative à la protection des personnes physiques à l'égard du traitement des données à caractère personnel et à la règlementation européenne (2016/679) relative à la protection des personnes physiques à l’égard du traitement des données à caractère personnel et à la libre circulation de ces données.

Le médecin investigateur a un devoir de confidentialité vis à vis des données collectées.

Ceci veut dire qu’il s’engage non seulement à ne jamais divulguer votre nom dans le cadre d’une publication ou d’une conférence mais aussi qu’il codera (que votre identité sera remplacée par un code d’identification dans l’étude) vos données avant de les transmettre au gestionnaire de la base des données collectées (service d’anesthésie, Grand Hôpital de Charleroi, Site Notre-Dame, Grand Rue 3 ; B-6000 Charleroi ; Belgique).

Le médecin investigateur et son équipe seront donc les seuls à pouvoir faire le lien entre les données transmises pendant toute la durée de l’étude et votre dossier médical.

Les données personnelles transmises ne contiendront pas d’association d’éléments qui permettraient de vous identifier.

Pour le gestionnaire des données de recherche désigné par le promoteur, les données transmises ne permettent pas de vous identifier.  Ce dernier est responsable de la collecte des données recueillies par tous les investigateurs participant à la recherche,  de leur traitement et de leur protection en conformité avec les impératifs de la loi belge relative à la protection de la vie privée.

Pour vérifier la qualité de l’étude, il est possible que votre dossier médical soit examiné par des personnes soumises au secret professionnel et désignées par le comité d'éthique, le promoteur de l’étude ou un organisme d’audit indépendant. En tout état de cause, cet examen de votre dossier médical ne peut avoir lieu que sous la responsabilité du médecin investigateur et sous la supervision d'un des collaborateurs qu’il aura désigné.

Les données de recherche (codées) pourront être transmises aux autorités réglementaires belges ou autres, aux comités d’éthique concernés, à d’autres médecins et/ou à des organismes travaillant en collaboration avec le promoteur.

Votre consentement à participer à cette étude implique donc aussi votre consentement à l’utilisation de vos données médicales codées aux fins décrites dans ce document d’information et à leur transmission aux personnes et instances susmentionnées.

Le promoteur utilisera les données collectées dans le cadre de l’étude à laquelle vous participez mais souhaite également pouvoir les utiliser dans le cadre d’autres recherches concernant la même maladie que la vôtre. Toute utilisation de vos données en dehors du contexte décrit dans le présent document ne pourrait être menée qu’après approbation du comité d’éthique.

Si vous retirez votre consentement à  participer à l’étude, afin de garantir la validité de la recherche, les données codées jusqu’au moment de votre interruption seront conservées.  Aucune nouvelle donnée ne pourra être transmise au promoteur.

***Assurance***

Toute participation à une étude clinique comprend un risque aussi petit soit-il.  Le promoteur assume, même en l’absence de faute, la responsabilité du dommage causé au participant (ou en cas de décès, à ses ayants-droit) et lié de manière directe ou indirecte à sa participation à la recherche. Le promoteur a souscrit un contrat d'assurance de cette responsabilité.

Vous êtes donc invité à faire part de tout problème de santé nouveau au médecin investigateur. Il pourra vous donner des informations complémentaires concernant les traitements possibles.

Si le médecin investigateur estime qu'un lien avec l'étude est possible (l'assurance ne couvrant pas l'évolution naturelle de votre maladie ni les effets secondaires connus de votre traitement habituel), il se chargera  d’informer le promoteur de l’étude qui se chargera d'initier la procédure de déclaration à l'assurance. Celle-ci nommera - si elle l'estime nécessaire - un expert pour juger du lien entre vos nouveaux problèmes de santé et l'étude.

En cas de désaccord soit avec le médecin investigateur, soit avec l'expert nommé par la compagnie d'assurances ainsi que chaque fois que vous l'estimeriez utile, vous ou - en cas de décès - vos ayants droit pouvez assigner l'assureur directement en Belgique (AMLIN, n° de police LXX057635).

La loi prévoit que la citation de l'assureur puisse se faire soit devant le juge du lieu où s'est produit le fait générateur du dommage, soit devant le juge de votre domicile, soit devant le juge du siège de l'assureur.

1. American College of Rheumatology [↑](#footnote-ref-1)
2. Electrocardiogramme [↑](#footnote-ref-2)
